# Mulberry leaf disease detection by CNN-ViT with XAI integration

**DOI:** 10.1371/journal.pone.0325188

**Published:** 2025-06-04

**Authors:** Mohammad Asif Hasan, Fariha Haque, Hasan Sarker, Rafae Abdullah, Tonmoy Roy, Nishat Taaha, Yeasin Arafat, Abdul Karim Patwary, Mominul Ahsan, Julfikar Haider

**Affiliations:** 1 Department of Electronics & Telecommunication Engineering, Rajshahi University of Engineering & Technology, Rajshahi, Bangladesh; 2 Department of Business Analytics and Data Science, Oklahoma State University, Stillwater, Oklahoma, United States of America; 3 Department of Data Analytics & Information Systems, Utah State University, Logan, Utah, United States of America; 4 Department of Electronics and Communication Engineering, Khulna University of Engineering & Technology, Khulna, Bangladesh; 5 School of Computer Science and Engineering, University of Electronic Science and Technology of China, Chengdu, China; 6 School of Electronic Science and Engineering, University of Electronic Science and Technology of China, Chengdu, China; 7 Department of Computer Science, University of York, Heslington, York, United Kingdom; 8 Department of Engineering, Manchester Metropolitan University, Manchester, United Kingdom; Instituto Politecnico Nacional, MEXICO

## Abstract

Mulberry leaf disease detection is vital for maintaining the health and productivity of mulberry crops. In this paper, a novel approach was proposed by integrating explainable artificial intelligence (XAI) techniques with a convolutional neural network (CNN) and vision transformer (ViT) for effective mulberry leaf disease classification with three disease classes. Initially, in this proposed CNN-ViT model, features are extracted using a customized CNN architecture, and then the extracted features are fed into ViT for leaf disease classification in a more streamlined approach. The CNN-ViT model achieved promising results with a projection dimension of 64, utilizing 8 heads and 8 transformer layers, yielding an accuracy of 95.60% with notable precision of 94.75%, recalls of 92.40%, and F1-scores of 93.45%. The proposed method also took 0.0017 seconds to predict an individual image. The accuracy of the proposed method was comparable to that of other state-of-the-art (SOTA) methods reported in the literature. Finally, Grad-CAM was utilized for detecting precise region of interest for diseased leaves, leaf spots, and leaf rust, providing interpretability and insights into the model’s decision-making process. This comprehensive approach demonstrates the effectiveness of explainable artificial intelligence (XAI) integration in the CNN-ViT model for mulberry leaf disease detection, paving the way for improved agricultural disease management strategies.

## 1. Introduction

Planting and breeding are combined in the sericulture industry [1]. One of the world’s first countries to create silk cocoons was China. Mulberry leaves are a major source of income for sericulture and a feeding source for silkworms [2]. Mulberry leaf number and quality are directly correlated with the ability of plants to create cocoons [3]. Mulberry trees are prone to several diseases that impair leaf development, lower leaf yields, and lower leaf quality. These factors negatively affect silkworm reproduction. The most prevalent mulberry leaf diseases are brown spot, powdery mildew, and red rust [4]. Like in any other industry, agricultural output helps farmers secure their financial future. For a powerful nation, expanding its agricultural sector is essential because it guarantees sustainability and fills a genuine need in the global economy. Like people, plants are prone to illness at different stages of life. Therefore, the farmer’s total crop yield and income consequently decline. As predicted, with more than 9 billion people on the planet by 2050, finding novel ways to detect and treat plant diseases can increase food production while lowering the need for pesticides [4]. Early identification of various disease types that can affect a crop is essential for ensuring the profitability of the agricultural industry. Conventional approaches have always relied on expert observation using only the naked eye to diagnose and detect plant diseases. Millions of small and medium-sized farmers worldwide find this strategy unsustainable due to its arduous, time-consuming, and costly nature. Consequently, there is a serious risk that unidentified diseases will infect other healthy plants.

In response to these challenges, scientists worldwide have developed automated systems that employ deep learning (DL) and machine learning (ML) methods to identify diseases in a variety of plants, including sunflower, watermelon, tomato, cotton, and rice [5]. The majority of the experiments were conducted using real-time data similar to those of plant and leaf images from the PlantVillage dataset. Additionally, the authors used 58 distinct plant species to categorize disorders affecting plants [6]. The Moraceae family includes mulberry, a deciduous woody tree species that grows quickly and is native to the Himalayan foothills of China and India [7]. Because silkworm larvae (*Bombyx mori*) eat the leaves of mulberry trees to produce mori silk, mulberry trees are economically valuable. Mulberries have long been used in animal husbandry and silk production, but their ecological significance has been underestimated. The importance of this plant has recently been widely acknowledged in other fields, including industry, medicine, and environmental safety [8]. In Bangladesh, sericulture has been recognized as a potential new economic engine. With support from the government and non-governmental organizations, Bangladesh has an enormous opportunity to achieve significant economic growth in this area. The Bangladesh government established the Bangladesh Sericulture Research and Training Centre in Rajshahi, a divisional-level city, to advance the field’s sericulture research. The People’s Republic of Bangladesh President signed Ordinance No. 62 in 1977, which established the Bangladesh Sericulture Board [9]. Originally, only two subdistricts (Bholahat and Shibganj) of the Rajshahi division’s present-day Nawabganj district were suitable for sericulture. Sericulture has spread to 36 of the 64 districts in Bangladesh since the country’s independence in 1971, helping to lessen poverty and improve job opportunities across the country, especially in rural regions [10].

However, feeding fresh mulberry leaves to silkworms is a prerequisite for successful sericulture. Mulberry plants are susceptible to various fungal diseases, the most common of which are leaf spot infections and Cercospora moricola leaf rust [10]. Mulberry plants are susceptible to many pests, such as hairy caterpillars. Pests and diseases usually result in a significant reduction in mulberry leaf yield, which in turn causes a decrease in silk production. Consequently, farmers suffer from massive financial losses, which negatively impact the overall economy of Bangladesh [11].

Moreover, silk gowns hold significant cultural value in certain regions of Bangladesh; hence, the use of diverse synthetic fibers has intensified the decline in silk production. If this issue is not resolved, those involved in this industry will lose their jobs, and future generations will not be aware of the traditional silk products and their aesthetic value.

Manual detection methods for mulberry leaf diseases are laborious and prone to error, whereas existing automated techniques, which are often based solely on convolutional neural networks (CNNs), struggle to capture holistic contextual information. Imbalanced datasets further hinder accuracy, with diseased leaves often being overlooked. The research motivation revolves around early detection of mulberry leaf diseases and development of appropriate prevention strategies to safeguard sericulture. The goal is to develop artificial intelligence techniques that help to mitigate crop loss on sericulture farms. Furthermore, the system highlights the affected regions on the diseased leaves through explainable AI.

This study builds upon the work presented in [10], where a CNN-based model was employed for mulberry leaf disease detection using data augmentation, which balanced the dataset. However, in real-life scenarios, the dataset is not always balanced. In contrast, the primary objective of this research is to develop a novel hybrid approach that integrates a CNN with a ViT to improve the model’s ability by capturing complex features from the dataset. Thus, while both studies focused on mulberry leaf disease detection, this paper presents an upgraded model with model complexity analysis and to achieve improved accuracy particularly with an imbalanced dataset, faster prediction and better interpretability.

The main contributions of this research to mulberry leaf disease prediction via the AI model are as follows:

1. A novel custom model that utilizes the strength of CNNs for feature extraction and extracted features fed into ViT for deep feature extraction and classification to handle the imbalanced nature of mulberry leaf datasets.
2. Different configurations of ViT with projection dimension, number of heads and transformer layers have been tested to optimise performance.
3. The performance of the proposed CNN-ViT model was compared with that of other transfer learning models (TLMs) combined with ViT to determine its efficacy.
4. With the proposed CNN-ViT model, the XAI technique was integrated by using Grad-CAM for visualizing and interpreting the decision-making process, enhancing model transparency and providing insights into the prediction process.
5. The performance of the CNN-ViT model was compared with that of the SOTA models..

The proposed strategy extracts initial features and reduces the dimensionality of plant disease images by using CNN and various transfer learning models, as opposed to applying the ViT model directly to raw images with all of their dimensions. The ViT model is then used to extract the deep features from the leaf images, emphasizing the interactions between all of the pixels that were collected in the previous phase. This strategy aims to mitigate time consumption while effectively leveraging both the CNN and ViT architectures for improved performance.

## 2. Related works

Recently, researchers have developed innovative methodologies for automating the detection and classification of a broad spectrum of plant diseases. Several of these methodologies are scrutinized and evaluated in this section.

Monalika et al. [2] presented a novel approach for mulberry infection detection using a CNN and You Look Only Once (YOLO). They trained the model for 10000 epochs for a total of 4000 images in 4 classes. There are 1000 images in each class. While the proposed model effectively identifies and classifies mulberry leaf diseases, the authors did not provide statistical performance metrics for the classification results. This omission raises concerns regarding the comprehensive evaluation of the model’s efficacy in disease classification.

Sandeep et al. [12] highlighted the potential of machine learning and image-processing techniques for identifying and diagnosing diseases affecting mulberry trees. The lack of detailed information about the model used in this paper is limited. Therefore, the ease of understanding the suggested methods makes it difficult for other researchers to replicate and validate them.

Himanshu et al. [13] proposed a capsule neural network approach that offered a novel alternative to traditional CNNs for the classification of mulberry leaves. They used 5,262 images in total consisting of 2 classes. Notably, the achieved accuracy of 82.04% was lower than that of the other methods. This highlights a potential limitation in the efficacy of capsule networks for this specific task. Despite the innovative approach outlined, the model’s performance suggests the need for further refinement and optimization to increase its accuracy and reliability in mulberry leaf classification. Additionally, to implement the capsule neural network, the authors utilized the VGG16 model, which demonstrates an insightful integration of existing architectures.

Thipwimon et al. [14] trained multiple CNN models, including MobileNetV1, MobileNetV2, NASNetMobile, DenseNet121, and Xception. They used 5,262 images in total consisting of 10 classes. The authors aimed to discover the best-performing model for leaf recognition. Notably, MobileNetV2 emerged as the top-performing individual model, achieving an accuracy of 91.19% on the mulberry leaf dataset. However, the authors further enhanced the performance by employing ensemble methods, such as unweighted average, weighted average, and unweighted majority vote, to combine the output probabilities of each CNN model. By esnsembling 5 CNN methods (5 pretrained models) without augmentation, they achieved an accuracy of 94.75%. However, while ensemble learning significantly improves classification accuracy, it can also introduce complexity and computational overhead, particularly in the training and inference phases, which may limit its scalability and practical applicability in resource-constrained environments.

Rakesh et al. [15] compared SVM and probabilistic neural networks (PNNs) for classifying plant diseases.. They used 200 images in total consisting of 4 classes. The SVM achieved 93.5% accuracy, and the PNN achieved 98% accuracy for 200 samples. The limitation of PNNs is their susceptibility to overfitting, particularly when dealing with smaller datasets or datasets with imbalanced class distributions. Owing to their complex architecture and tendency to model noise in the training data, PNN may exhibit high variance and poor generalizability on unseen data. Additionally, the computational complexity of PNN can be relatively high, especially when dealing with large-scale datasets, which may pose challenges in terms of training time and resource requirements.

Anusha et al. [16] used traditional ML algorithms. These methods are generally simpler and easier to implement but they might not be as effective as deep learning methods for complex image classification tasks.

Nahiduzzaman et al. [10] also achieved an accuracy of 95.05%, but they converted an imbalanced dataset into a balanced dataset. In real-life scenarios, each class is not evenly distributed. This is the main drawback of their model, which may not work well on imbalanced datasets.

Salam et al. [17] used the pretrained models MobileNetV3 Small, ResNet-50 and VGG-19. They achieved 96.4% accuracy with MobileNetV3 Small and 94.4% accuracy with ResNet50 and VGG-19. They also used TFLite for real-time user experience and Grad-Cam for model explainability. While highly accurate, the use of pretrained models can sometimes limit flexibility in adapting to new or more complex tasks.

Wen et al. [18] proposed a multi-scale residual network fusion SENet for the classification of mulberry leaf diseases, achieving an impressive accuracy of 98.72%. The model was trained on a dataset consisting of four classes. By incorporating multi-scale convolution kernels and the Squeeze-and-Excitation Networks (SENet) attention mechanism, the model significantly improved recognition performance, with enhanced precision, recall, and F1 scores. However, the increased network depth introduces a potential drawback, as it may require more computational resources and poses a risk of overfitting if not properly managed.

The existing SOTA methods for mulberry leaf disease detection focus on achieving improved accuracy but often overlook crucial aspects such as easy interpretation by Grad-CAM and the inability to handle imbalanced datasets, which is very common in real-life scenarios. Furthermore, extracting high-level semantic and contextual features from the image by capturing relationships between individual image patches through self-attention, making it highly effective in learning complex visual patterns that is generally missing in SOTA studies. Therefore, the aim of this work was to develop a more robust model that can extract complex features, handle imbalance dataset, accurate and fast classification of different diseases associated with the mulberry leaves and visually identifying region of interest (ROI) through an appropriate XAI technique.

## 3. Methodology

### 3.1. Overall model architecture

**Fig 1:**
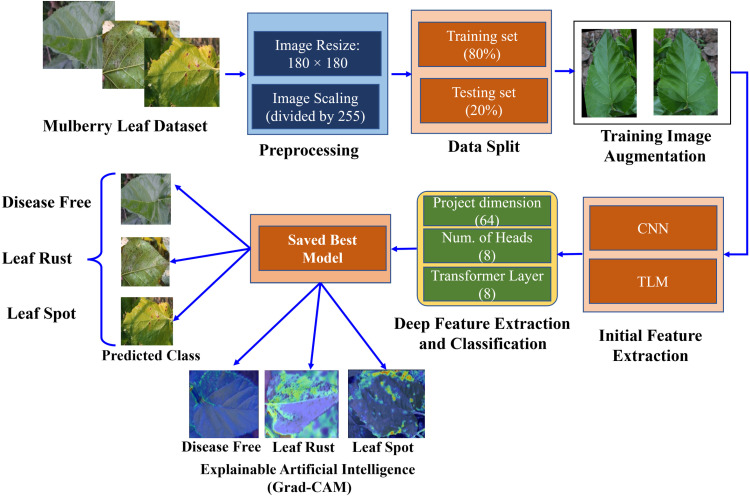
Proposed workflow for classifying mulberry leaf diseases.

Fig 1 illustrates the proposed workflow for classifying mulberry leaf diseases. It starts with preprocessing the mulberry leaf dataset by resizing and scaling the images. The dataset is split into training (80%) and testing (20%) sets, with the training images further augmented. Initial feature extraction is carried out using a CNN and TLM, followed by deep feature extraction and classification with a ViT. The best-performing model was saved and used to predict leaf disease classes: disease-free, leaf rust, or leaf spot. Grad-CAM is employed for model interpretability, providing visual explanations by highlighting the crucial areas in the leaf images that influence the model’s predictions. This workflow demonstrates a comprehensive approach to classifying mulberry leaf diseases using advanced CNN-ViT, combining a CNN for feature extraction and ViT for classification, along with Grad-CAM for model interpretability.

Segmentation was not employed in this study because segmentation typically requires precise boundary identification and localization of disease-affected areas, which can be computationally expensive and prone to inaccuracies. For mulberry leaf diseases, such as rust and spots, diseased regions often exhibit subtle variations in color, shape, and texture, making it difficult to segment affected areas accurately. Inaccurate segmentation can lead to incorrect classification, further affecting the model’s overall performance. Since our goal is to develop a system that can operate efficiently in classifying agricultural applications, segmentation would introduce unnecessary computational overhead, making the model less feasible for real-world deployment, especially in resource-constrained environments. Finally, segmentation followed by classification may introduce additional stages in the workflow, which can lead to compounding errors.

The proposed CNN-ViT hybrid approach focuses on global feature extraction. ViT has the ability to capture both local and global contexts from images, making it highly effective in detecting diseased regions by using Grad-CAM as part of the XAI component. The regions that contribute to the classification can be visualized, effectively addressing the need for interpretability that segmentation aims to achieve.

### 3.2. Dataset description

The images used in this research were made publicly available during a previous study [10]. Images of leaves from mulberries are usually taken using digital cameras or cameras on smartphones. These images could be from labs, greenhouses, or even different regulated situations, such as natural habitats. Leaf spot and leaf rust, two prevalent and authorized mulberry leaf diseases, were selected for the study after experts affiliated with the Bangladesh Sericulture Development Board (BSDB) in Rajshahi provided necessary advice.

**Fig 2:**
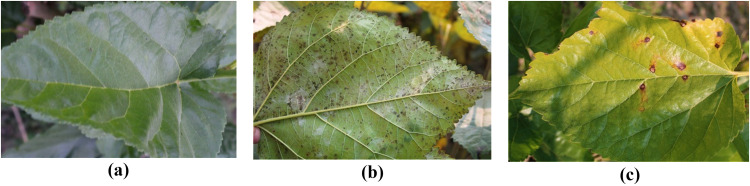
Three classes of the mulberry leaf dataset: (a) disease-free leaves, (b) leaf rust (c) and leaf spot.

Images of mulberry gardens in Mirganj, Bagha, Rajshahi, and Vodra, Rajshahi, were collected as described in a previous study. To ensure realism, high-resolution DSLR cameras were used to take these images in actual environments. The mulberry dataset comprises a comprehensive collection of 1,091 images meticulously categorized by a sericulture specialist into three distinct classes: 440 leaves exhibiting healthy characteristics, 489 leaves displaying leaf rust, and 162 leaves exhibiting leaf spots [16]. Fig 2 shows the three classes that were used in this research. The resolution of each leaf image is 4,000 × 6,000 pixels.

### 3.3. Dataset preprocessing and augmentation

The quality of image preprocessing has a significant effect on the classification accuracy. The goal of this work is to streamline the image-processing stages so that embedded systems can integrate them with ease. The images within the dataset were resized to 180 × 180 pixels during preparation. The amount of additional storage space and processing power required is reduced by this downsizing strategy. Images usually use many intensity levels to be represented. After the pixel values were divided by 255, normalization was used to simplify the images and to change the scale from 0–255–0–1.

**Fig 3:**
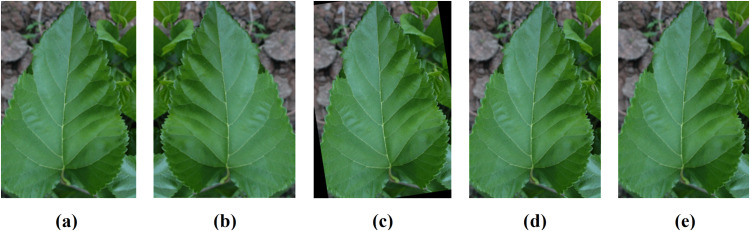
(a) Original image and augmented images: (b) horizontal flip (c) rotation, (d) zoom by width factor, (d) zoom by height factor.

In the data augmentation process utilized in this study, a combination of techniques is applied to enrich the diversity and robustness of the training dataset. A random rotation of 20° was used, as shown in Fig 3(c). The images were flipped in the horizontal orientation, as shown in Fig 3(b). In addition, a zoom in width and height (scale of 0.2) was used, as shown in Fig 3(d) and 3(e).

Collectively, these augmentation strategies contribute to enriching the training data, facilitating the model’s ability to learn robust features and patterns and ultimately improving its performance in mulberry leaf disease detection tasks. Fig 3 presents a few examples of image augmentations. Table 1 presents the distribution of mulberry leaf data according to disease classes.

**Table 1:** Data distribution in each class for the training and testing sets.

Classes	Training images (pre-augmentation)	Training images (post-augmentation)	Testing images
Healthy	352	1760	88
Leaf Rust	392	1960	97
Leaf Spot	130	650	32

### 3.4. CNN-ViT

**Fig 4:**
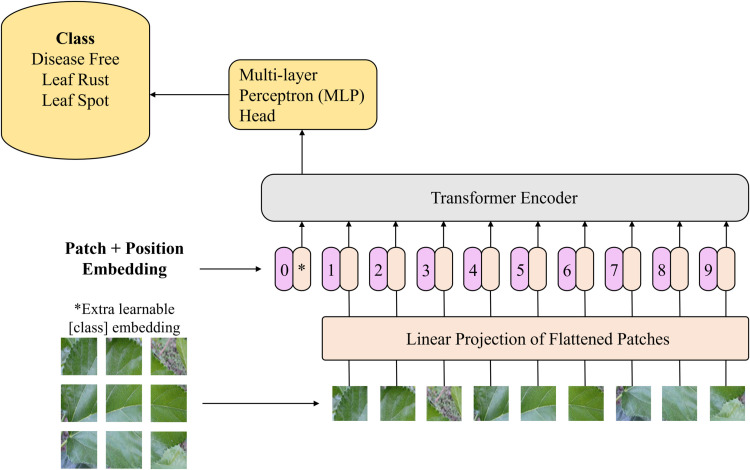
Traditional ViT for leaf image classification.

The proposed CNN ViT model architecture encompasses the integration of CNNs and transformers to enhance the performance of visual recognition tasks [19]. Fig 4 presents the traditional Vision Transformer (ViT) architecture for leaf image classification, detailing the patch embedding process, positional encoding, transformer encoder, and classification head. It serves as a baseline to highlight the differences of the proposed CNN-ViT hybrid approach in capturing both local and global features for improved classification accuracy. The first phase in this methodology contains the input of an image into the model, which subsequently undergoes processing through the CNN component, leading to the generation of a feature map. After the feature map is partitioned into patches, it is flattened and then used as input for the ViT model. The ViT model receives these patches as a progression of tokens and utilizes a succession of transformer blocks to represent spatial and channelwise connections between the patches. The sequence that has been encoded is directed through a linear classifier, resulting in the production of ultimate predictions.

#### 3.4.1. Initial feature extraction.

**Fig 5:**
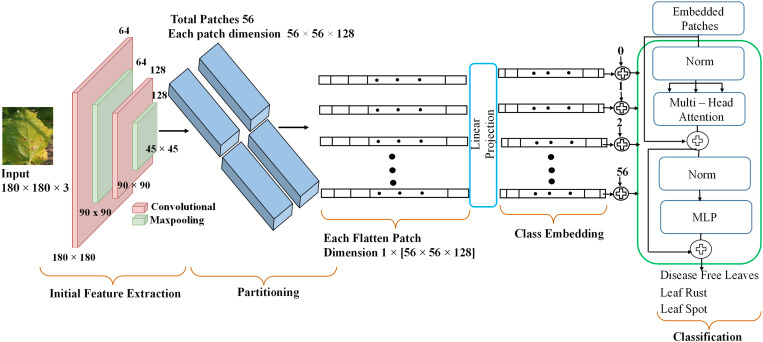
Proposed architecture of the CNN-ViT model.

In this study, a comprehensive approach to feature extraction was employed by utilizing well-established transfer learning architectures, including VGG16, VGG19, ResNet50, Xception, MobileNetV2, and custom CNNs, as initial feature extractors. Following established practices, in transfer learning, the fully connected layers are removed from these architectures to focus solely on feature extraction capabilities. Additionally, to prevent overfitting and promote efficient training, the layers of the base networks were frozen during the training process. This strategy ensured that only the newly added layers, which are responsible for task-specific learning, were trainable, thereby enhancing the generalization ability of the models. These pretrained CNN and custom CNN architectures, with modified configurations, aimed to capitalize on their learned representations while customizing the models to suit the specific requirements of visual recognition tasks. The final dimensions of the initial features extracted by the TL models, e.g., VGG16, VGG19, ResNet50, Xception, and MobileNetV2, are 5 × 5 × 512, 5 × 5 × 512, 6 × 6 × 2048, 6 × 6 × 2048, and 6 × 6 × 1280, respectively. The final dimensions of the initial features extracted by the custom CNN are 45 × 45 × 128. The ViT model then receives these features as inputs and processes them in accordance with the procedures illustrated in Fig 5.

#### 3.4.2. Deep feature extraction.

The kernel-based texture features are extracted from the CNN-based model, and then the extracted features are fed into ViT; thus, the CNN-ViT [20] model is constructed. The ViT operates by using the attention mechanism to establish connections between adjacent pixels as well as pixels located at a considerable distance. To carry out the operations of the attention mechanism, the input image is first divided into discrete patches. The above process is similar to a convolutional layer with a kernel, and the output is a four-dimensional matrix with batch indexing, with rows, columns, and depths making up the other three dimensions.

The image’s shape $I\in {\mathbb{R}}^{H\times W\times C}$ is then reshaped into $PP\in {\mathbb{R}}^{N\times P^{2}\times C}$, where C represents the number of channels and where H and W represent the width and height of the image, respectively. Conversely, N represents the total number of patches determined as [21]:


$$\begin{array}{c} N=\frac{H\times W}{P^{2}} \end{array}$$ (1)

where P is the patch size. The input feature size for the custom CNN is 45 × 45, and the patch size is 6 × 6. The number of patches is calculated as follows:

$\left(\frac{H\times W}{p^{2}}\right)=\frac{45\times 45}{(6{)}^{2}}=56(almost)$ After patch partitioning, the raw image I is converted into a 2D matrix, PP, and linearly projected into a 1D embedding vector, $PP_{\text{LinearProjection }}$, with dimensions of 64 [21].


$$PP=[\begin{array}{cccc} \left[I_{1}\right] & \left[I_{2}\right] & \ldots & \left[I_{8}\right]\\ \left[I_{9}\right] & \left[I_{10}\right] & \ldots & \left[I_{16}\right]\\ \ldots & \ldots & \ldots & \ldots \\ \left[I_{49}\right] & \left[I_{50}\right] & \ldots & \left[I_{56}\right] \end{array}]$$ (2)

$$PP_{\text{LinearProjection }}=[\begin{array}{cccc} {}[I_{1}^{1}\; & I_{1}^{2} & \ldots & \;I_{1}^{64}]\\ {}[I_{2}^{1}\; & I_{2}^{2} & \ldots & \;I_{2}^{64}]\\ \ldots & \ldots & \ldots & \ldots \\ {}[I_{56}^{1}\; & I_{56}^{2} & \ldots & I_{56}^{64} \end{array}]$$ (3)

The patches are embedded using spatial embedding since the transformer has a high computational cost. During this embedding process, the image patches are first grouped into smaller groups before being applied to larger image sizes [22]. The embedding position $E_{POS}$ technique is implemented by combining sine and cosine functions with varying frequencies [23]. The cosine function is utilized when the patch is positioned oddly. On the other hand, the sine function is employed when the patch is in an even position. Here, location is indicated by the word pos, whereas dimension is indicated by i. The sinusoid has many encoded locations that hold the whole positional embedding. The maximum length of the patch group is represented by the variable d. After that, a positional embedding is joined with the linear projected patch to create an embedded patch [21].


$$\begin{array}{c} E_{\text{POS }}=\left\{ \begin{array}{c} \sin\left(\frac{pos}{1000^{\frac{2i}{d}}}\right),i\text{ is even }\\ \cos\left(\frac{pos}{1000\frac{2i}{d}}\right),i\text{ is odd } \end{array}\right.\; \end{array}$$ (4)

$$EP=concatenate\left(PP_{{\text{Linear }}_{\text{Projection }}},E_{POS}\right)$$ (5)

The embedded patch is thereafter transmitted to the encoded block following the linear projection and positional embedding processes. The encoder consists of four identical blocks, with each block comprising a cluster of six layers. These layers include a normalization layer, followed by a multi-head attention (MHA) layer and a multi-layer perception (MLP). Initially, the input of the encoder block, denoted EP, is combined with the output of the multiple-source analyzer. The output is subsequently sent toward a normalization layer, whereas the MLP incorporates a dense dropout layer. The attention output is concatenated with a skip connection from the input, hence enhancing the influence of position. This is achieved by providing the following layer with the original embedded patch. Attention is computed by utilizing three embedding matrices: key K, query Q, and value V, where the matrices are calculated using weight matrices $W_{Q}$, $W_{K}$ and $W_{V}$ by using the

The following equations [20] are used in the MHA mechanism:


$$\text{Query, }Q=EP.W_{Q}$$ (6)

$$Key,K=EP.W_{K}$$ (7)

$$\text{Value, }V=EP.W_{V}$$ (8)

where EP is the embedded patch, and the weight matrices are $W_{Q}$, $W_{K}$, and $W_{V}\in {\mathbb{R}}^{d_{\text{model }}\times d_{k}}$.

The single attention function, referred to as a head, is carried out using the following equation. This operation is executed in parallel multiple times within the Multi-Head Attention (MHA) layer, where the attention is computed using Equation 9. In this case, the dot-scaled product $d_{K}$ keeps the attention value from skyrocketing. The correlation between two visual patches is represented by this attention value, which also serves as a scoring function. Since there are four heads in the MHA in the suggested framework, the representation is as follows [21]:


$$\text{Attention, }(Q,K,V)=softmax\left(\frac{QK^{T}}{\sqrt{d_{k}}}\right)V$$ (9)

$$\text{MHA = Attention, }(Q,K,V{)}_{\times 8}$$ (10)

The MLP, which employs a dense layer with Gaussian error linear unit GELU activation in the later layers, provides nonlinearity in this process. where φ is the cumulative distribution of the Gaussian distribution. Finally, the output of the layer is removed, which is $Transformer_{\text{featureShape}}$, and later, we flatten it accordingly.


 GELU (x)=xP(X≤x)=xφ(x) (11)

$${\text{Transformer }}_{\text{featureShape }}=GELU(MHA)$$ (12)

$$Y_{\text{Transformer }}=\text{ flatten }\left({\text{ Transformer }}_{\text{featureShape }}\right)$$ (13)

The deep neural network classifier then receives $Y_{\text{Transformer }}$.

#### 3.4.3. MLP classifier.

The classifier employed in the proposed model is the MLP head. Since the encoder consists of four blocks, after the fourth iteration of the encoder, the deep features are obtained. After generating the deep features in the preceding stage, the transformer encoder inputs them into the classifier. This determines the


$$\;P(j)=MLP_{\text{head }}\left(X_{i}^{\prime }(4)\right);j=1,\cdots 4$$ (14)

classification of the image. The MLP classifier receives the deep feature vectors $X_{i}^{\prime }(4).$ as input values. The input layer consists of 128 neurons, which is equal to the dimension of the deep feature vector. $X_{i}^{\prime }(8).$ Two concealed layers are employed, with dimensions of 2048 and 1024 for the initial and subsequent levels, respectively. Finally, the output of the MLP classifier consists of 3 neurons, which represent the total number of classes in the dataset.

#### 3.4.4. Proposed CNN architecture.

In this research, the CNN model was used for initial feature extraction for mulberry leaf disease detection, and a sequential architecture comprising multiple layers aimed at extracting and learning relevant features from input images was employed. The initial layers consist of convolutional operations with 64 and 128 filters, each followed by ReLU activation functions to introduce nonlinearity and enhance feature representation. By training multiple models consisting of different convolutional layers with different configurations and evaluating their performance, the optimal architecture that achieves the best balance between accuracy and computational complexity is determined. Subsequently, max pooling layers were incorporated to downsample the feature maps, reducing the computational complexity while preserving important spatial information. The resulting feature maps were then flattened into a one-dimensional array before passing through a dropout layer, aiding in regularization by randomly deactivating a portion of neurons during training to prevent overfitting.

### 3.5. Explainable AI (Grad-CAM)

Grad-CAM stands out as a powerful technique for unraveling the decision-making processes of deep neural networks, with a primary focus on CNNs employed in image classification. It serves as an enhancement over the class activation mapping (CAM) method, broadening its applicability beyond global average pooling layers. Grad-CAM operates by computing gradients associated with the target class during the backward pass, specifically with respect to the feature maps of the final convolutional layer. These gradients guide the determination of importance weights for each feature map through global average pooling. The subsequent linear combination of the original feature maps, weighted by these importance scores, undergoes rectified linear unit (ReLU) activation to accentuate positive contributions. The resulting heatmap, obtained by upsampling this activated map, vividly illustrates the pivotal regions in the input image that significantly influence the model’s classification decision. This interpretability is particularly valuable in fields such as medical imaging and autonomous vehicles, where understanding the neural network’s decision-making process is paramount. Grad-CAM has emerged as a widely embracing tool for enhancing transparency and interpretability in the intricate workings of CNNs [24]. Grad-CAM has the following three basic steps:

1. Class score computation:

The gradients of the class score $y^{c}$ are calculated in relation to the feature mappings $A^{k}$ of the last convolutional layer that remains before the classifier.


$$\frac{{\partial y}^{c}}{{\partial y}^{k}}{\in R}^{F\times U\times V}$$ (15)

$${A^{k}\in R}^{F\times U\times V}$$ (16)

1. Gradient computation:

The global average pools the gradients over the width (indexed by i) and height (indexed by j) to produce the attention weights$\;\alpha^{c}$.


$$\mathrm{\backslash [}\alpha_{k}^{c}=\frac{1}{Z}\sum_{i}\sum_{j}\frac{\partial y^{c}}{\partial A_{ij}^{k}}=\in R^{F\times 1\times 1}=\in R^{F}\mathrm{\backslash ]}$$ (17)

1. Weighted Combinations and Heatmap Generation:

Applying the ReLU (.) function to the final Grad-CAM heatmap to preserve only the positive values and transform each of the negative values into zero. The heatmap is generated by the weighted ($\alpha^{c}$) sum of feature maps ($A^{k}$).


$$\mathrm{\backslash [}H_{heatmap}^{c}=RELU\sum_{k}\alpha_{k}^{c}A^{k}=\in R^{U\times V}\mathrm{\backslash ]}$$ (18)

These equations represent the key steps of the Grad-CAM algorithm, where the gradients of the class score with respect to the feature maps are computed, weighted combinations are performed, and a heatmap is generated. The resulting Grad-CAM heatmap highlights the important regions in the input image that contribute to the prediction of the target class.

### 3.6. Experimental configurations

Achieving high accuracy with the proposed model requires meticulous tuning of its hyperparameters. Table 2 presents the values of these hyperparameters. The observations indicate that the Adam optimizer achieves faster learning rates than other popular optimizers do. Additionally, dropout layers have been incorporated to mitigate the risk of overfitting.

**Table 2:** Hyperparameter configurations used in this work.

Hyperparameters	Value
Batch size	16
Optimizer	Adam
Initial learning rate	0.001
Epochs	50
Weight decay	0.0001
Label smoothing	0.1
Loss function	Sparse Categorical Cross entropy

A balance between memory consumption and training speed influences practical decisions regarding batch size. In our methodology, a batch size of 64 was employed for normalization. Following numerous experiments conducted with training data from the combined dataset, the remaining hyperparameter values were selected somewhat arbitrarily. A trial-and-error methodology was employed to iteratively fine-tune the hyperparameters for optimal performance. Table 3 presents data associated with system configuration used during the model development.

**Table 3:** System configuration of the proposed model.

Tools	Configuration
Programming Language	Python
Backend	Keras with TensorFlow
Disk Space	78.2 GB
GPU RAM	15 GB
GPU	Nvidia Tesla T4
System RAM	12.72 GB
Operating system	windows 11
Input	Mulberry Leaf
Input Size	180 x 180

### 3.7. Performance metrics

The first step involved partitioning the dataset into two distinct subsets, the training set and the testing set, maintaining an 80:20 ratios. Various evaluation metrics were computed to compare and contrast the performance of the proposed CNN model. To assess model performance, a confusion matrix was generated based on the model predictions. Evaluation metrics such as accuracy, F1 score, precision, recall, and AUC were calculated to differentiate between different models [25]. The following metrics were derived by the established methodologies.


$$Accuracy=\frac{TP+TN}{TP+FP+TN+FN}$$ (19)

$$Precision=\frac{TP}{TP+FP}$$ (20)

$$Recall=\frac{TP}{TP+FN}$$ (21)

$$\text{F1 }-\text{ score }=\frac{2\times \text{ precision }\times \text{ recall }}{\text{ precision }\times \text{ recall }}$$ (22)

$$AUC=\frac{1}{2}\left(\frac{TP}{TP+FN}+\frac{TN}{TN+FP}\right)$$ (23)

Here, FP = false positive, TP = true positive, FN = false negative, and TN = true negative. TP indicates when the model correctly identifies a diseased leaf as diseased. FP indicates when the model incorrectly predicts a healthy leaf as diseased. FN indicates when the model incorrectly predicts a diseased leaf as healthy. TN indicates when the model correctly identifies a healthy leaf as healthy.

## 4. Result analysis & discussion

### 4.1. Optimum parameter selection

Table 4 presents the selected optimum parameters of the CNN-ViT model for image classification tasks. obtained from the project dimensions, number of heads, and number of transformer layers of 64, 8, and 8, respectively. With an image size of 180 and a patch size of 6, the model divides the input image into 56 patches from equation (1). The projection dimensions of 64 and 8 heads allow for expressive feature representation and diverse relationships to be captured within the data. The transformer unit is specified as 128 for the encoder and 64 for the decoder, with 4 encoder layers.

**Table 4:** Selected optimum parameters of CNN-ViT.

Parameters	Values
Image Size	180
Patch Size	6
Number of Patch	56
Projection Dimension	64
Number of Heads	8
Transformer Unit	128, 64
Number of transformer layers	8
MLP Head Unit	2048,1024

### 4.2. Results analysis of CNN-ViT

Table 5 shows the performance analysis of CNN-ViT with different configurations, specified by the projection dimension and the number of heads and transformer layers. Among the configurations, 64-8-8 yields the highest accuracy at 95.60% and precision at 94.75%, resulting in an F1 score of 93.45%.

**Table 5:** Performance analysis of CNN-ViT with different configurations.

Projection dimension-Num of Heads-Transformer Layers	Accuracy	Precision	Recall	F1-score	Prediction Time for single image (seconds)
64-8-4	93.60	89.72	90.87	90.26	0.0016
64-4-4	94.06	92.40	89.56	90.81	**0.0015**
64-4-8	94.06	**95.69**	87.04	90.02	0.0016
64-8-8	**95.60**	94.75	**92.40**	**93.45**	0.0017

Note: bold values indicate best results

**Fig 6:**
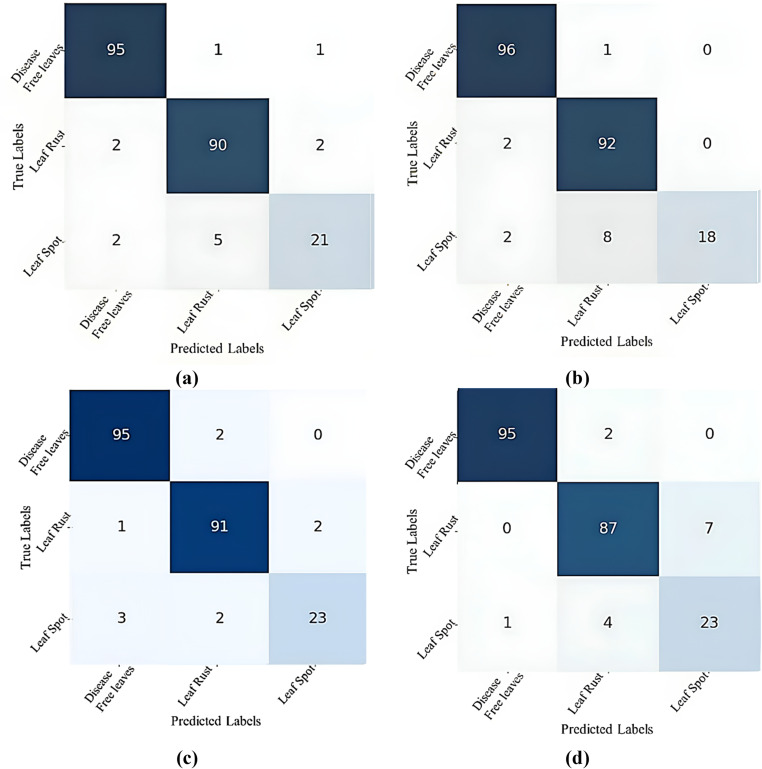
Confusion matrix for different CNN-ViT configurations (projection dimension-number of heads-transformer layers): (a) 64-4-4, (b) 64-4-8, (c) 64-8-8 and (d) 64-8-4.

Fig 6 displays the confusion matrix of the CNN-ViT model for mulberry leaf disease classification across various configurations: (a) 64-4-4, (b) 64-4-8, (c) 64-8-8, and (d) 64-8-4. An insightful analysis of these matrices revealed significant findings about the performance of different configurations. Notably, the confusion matrix for the 64-8-8 configuration shows that this setup has excelled in making accurate predictions. The analysis highlights the model’s ability to minimize false positives, indicating a high level of precision in its predictions. When the true label was disease-free leaves, this configuration correctly predicts 64-8-8 disease-free leaf instances 95 times. When the true label was disease-free leaves only 2 times, it predicted a false result. Again, this configuration correctly identified 91 instances of leaf rust. When the true label was leaf rust, only 3 false results were predicted; again, for the leaf spots, only 5 false results were predicted, and 23 correct results were predicted.

**Fig 7:**
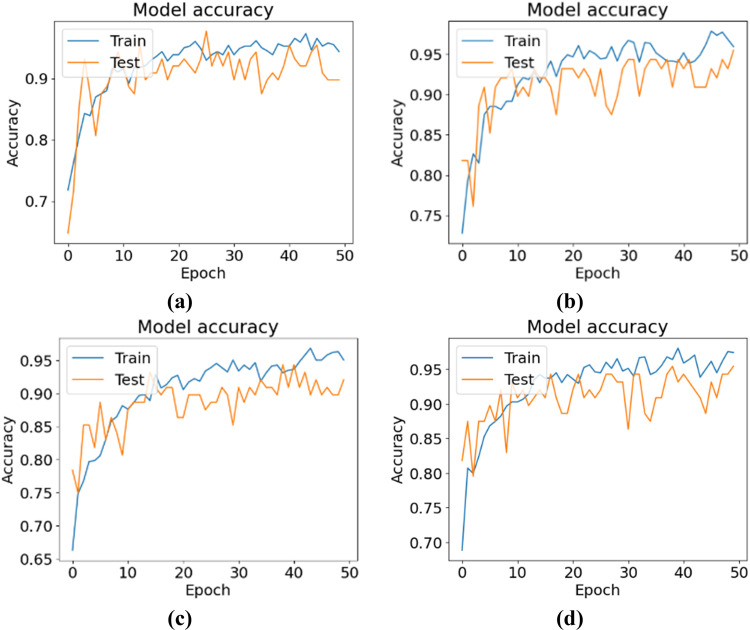
Accuracy curves for different CNN-ViT configurations (projection dimension-number of head-transformer layers): (a) 64-4-4, (b) 64-4-8, (c) 64-8-8 and (d) 64-8-4.

**Fig 8:**
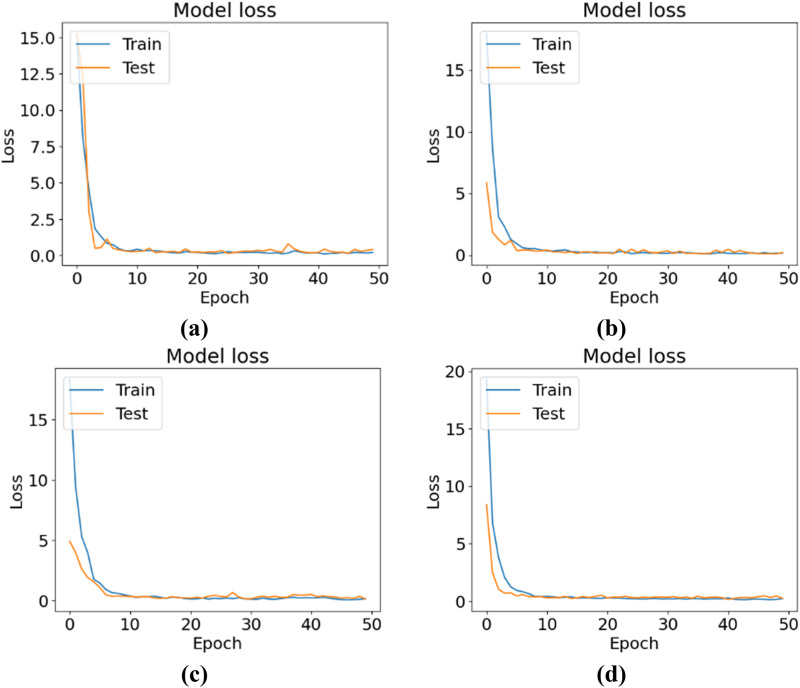
Loss curves for different CNN-ViT configurations (projection dimension-number of head-transformer layers): (a) 64-4-4, (b) 64-4-8, (c) 64-8-8 and (d) 64-8-4.

**Fig 9:**
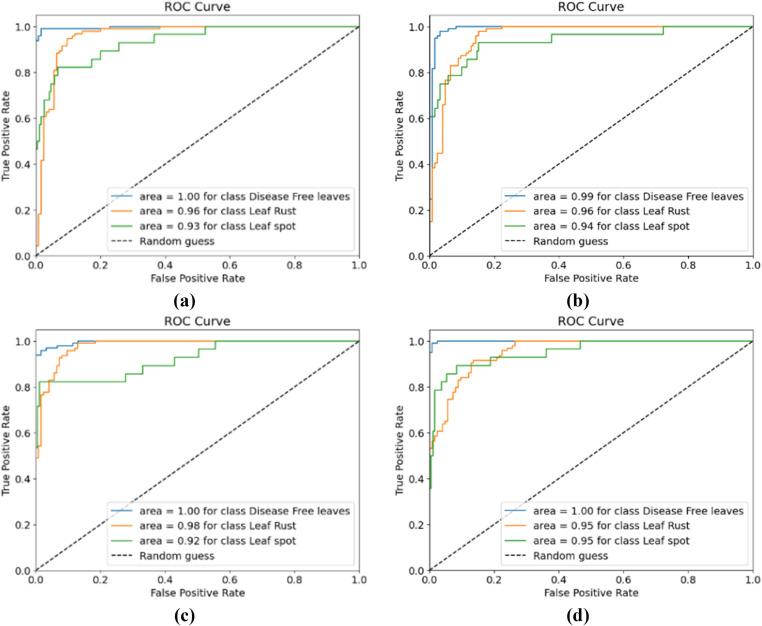
ROC curves for different CNN-ViT configurations (projection dimension-number of head-transformer layers): (a) 64-4-4, (b) 64-4-8, (c) 64-8-8 and (d) 64-8-4.

Fig 7 shows the training and testing accuracy curves, and Fig 8 shows the training and testing losses for all the experimental configurations. According to the accuracy and loss curves, the performances of the different configurations of the CNN-ViT model on the mulberry leaf dataset can be understood. Fig 7(c) shows the accuracy curve for the 64-8-8 configuration, which produces the highest testing accuracy of 95.43%. Fig 9 shows the ROC curve that was generated for each configuration and shows that for the 64-8-8 configuration, an AUC of 1 is achieved for disease-free leaves, 0.98 for leaf rust and 0.92 for leaf spots, which are the best results.

**Fig 10:**
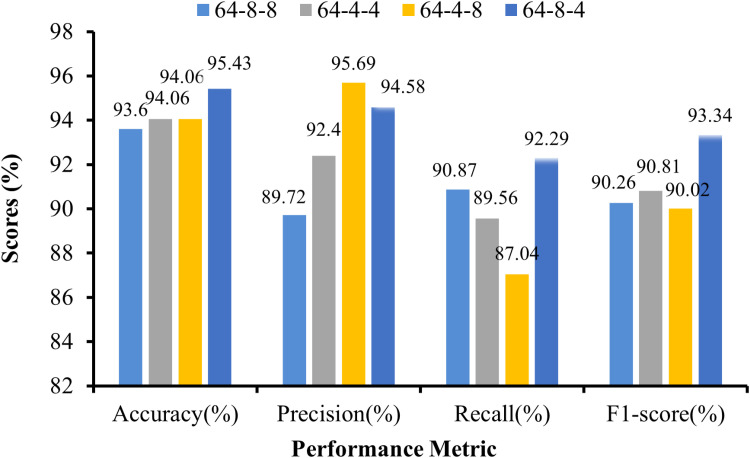
Graphical representation of CNN-ViT performance metrics.

Fig 10 presents a graphical representation of the performance metrics of the CNN-ViT model for different combinations of projection dimension-number of head-transformer layer configurations. This higher accuracy and precision can be attributed to the increased dimensionality of the projections (64), which allows for more expressive representations, a greater number of heads (8), and a transformer layer of 8, which enables the model to capture more diverse features and relationships within the data. The results highlight the impacts of varying projection dimensions, the number of heads, and the number of transformer layers on the performance of the CNN-ViT model. These findings offer valuable insights for optimizing model configurations to achieve superior performance in image recognition tasks, balancing the CNN-ViT configuration for computational efficiency with accuracy.

Table 6 shows the 5-fold performance analysis of the proposed CNN-ViT model. Each fold in the 5-fold cross-validation exhibits consistent performance, with accuracy, precision, recall, and F1 score remaining within a narrow range. The results demonstrated stability across all the folds, with no drastic variations between the lowest (Fold-1) and highest (Fold-5) accuracy values. This consistency proves that the model generalizes well across different subsets of the data, avoiding overfitting or underfitting on specific folds. The gradual increase in performance, especially for Fold-4 and Fold-5, also suggested that the model maintained robustness and performed reliably on unseen data, further confirming its generalizability.

**Table 6:** Fivefold analysis of the proposed CNN-ViT model.

No. of Folds	Accuracy	Precision	Recall	F1-score
Fold-1	94.80	94.10	92.15	92.80
Fold-2	95.10	94.25	92.30	93.05
Fold-3	95.25	94.45	92.50	93.10
Fold-4	95.43	94.58	92.29	93.34
Fold-5	95.60	94.75	92.40	93.45
Avg.	**95.24**	94.43	92.33	93.15

Additionally, the number of MLP head units is set to 2048 for the first layer and 1024 for the second layer. These parameters are carefully tuned to optimize model performance while ensuring efficient utilization of computational resources. This selection process emphasizes the importance of parameter optimization in achieving optimal performance in image recognition tasks, contributing to advancements in the field.

**Fig 11:**
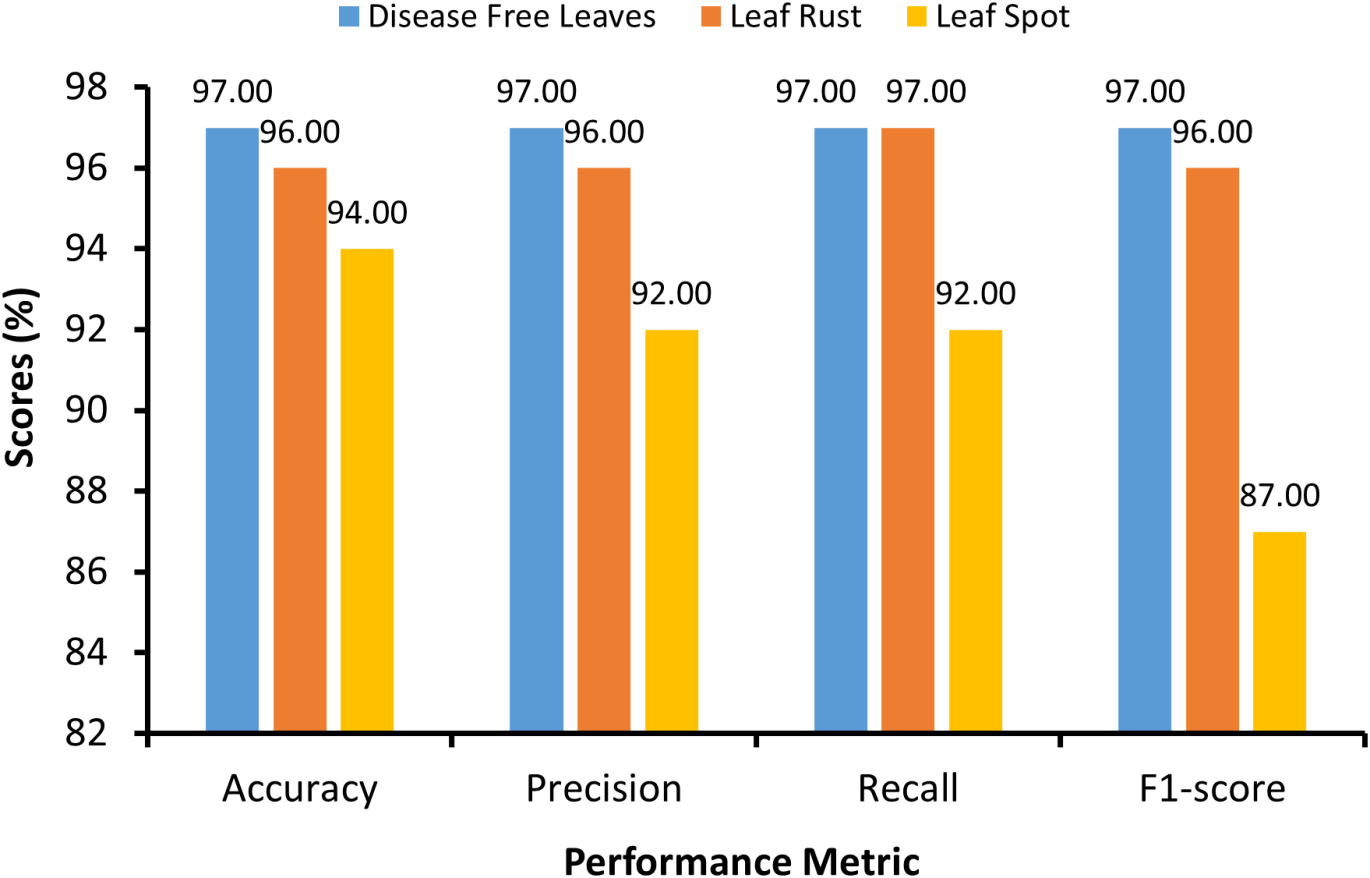
Graphical representation of the class wise performance metrics of the proposed model.

Table 7 and Fig 11 present class wise performance metrics of the proposed model for leaf disease classification. The results demonstrate high precision, recall, and F1-scores for the disease-free leaf and leaf rust classes, indicating the accurate classification of these classes. However, the class “Leaf Spot” results in slightly lower performance metrics, particularly in terms of the recall and F1 score, potentially because fewer instances exist in the dataset. Overall, the proposed model shows promising performance in classifying leaf diseases, with specific areas for potential refinement or further investigation to increase classification accuracy across all classes. While performance metrics offer valuable insights into model accuracy, relying solely on these results may not provide a comprehensive understanding of model behavior. Therefore, to enhance model interpretability and confidence, Grad-CAM was employed. Grad-CAM is a technique that visualizes the regions of input images that are most influential in making predictions, thereby providing insights into the decision-making process of the model.

**Table 7:** Performance metrics of the proposed model based on individual classes.

Disease class	Accuracy	Precision	Recall	F1-score
Disease Free leaves	97	96	98	97
Leaf Rust	96	96	97	96
Leaf spot	94	92	92	87

### 4.3 Model complexity analysis

In Table 8, unlike traditional ViTs, which require large datasets and high computational resources, the CNN-ViT model significantly reduces trainable parameters (3.2 M) and model size (12.8 MB) while maintaining high accuracy (95.60%). This efficiency is achieved by leveraging CNN for initial feature extraction, which reduces redundant computations before feeding refined features into ViT, making it more effective in handling imbalanced datasets compared to traditional ViT models. Additionally, the CNN-ViT model is lightweight, less complex and computationally efficient, making it ideal for real-world deployment on edge devices, for real-time mulberry leaf disease detection.

**Table 8:** Comparison of trainable parameters and model sizes of ViT variants and the proposed CNN-ViT model.

Model	Trainable Parameters (M)	Model Size (MB)	Patch Size	Hidden Dimension (D)	Heads (H)	Layers (L)
ViT-Ti (Tiny)	5.7M	~22.8MB	16 × 16	192	3	12
ViT-S (Small)	22M	~88MB	16 × 16	384	6	12
ViT-B (Base)	86M	~344MB	16 × 16	768	12	12
ViT-L (Large)	307M	~1.2GB	16 × 16	1024	16	24
ViT-H (Huge)	632M	~2.5GB	14 × 14	1280	16	32
Proposed CNN-ViT	3.2M	~12.8MB	6 × 6	64	8	8

### 4.4. Ablation study

**Fig 12:**
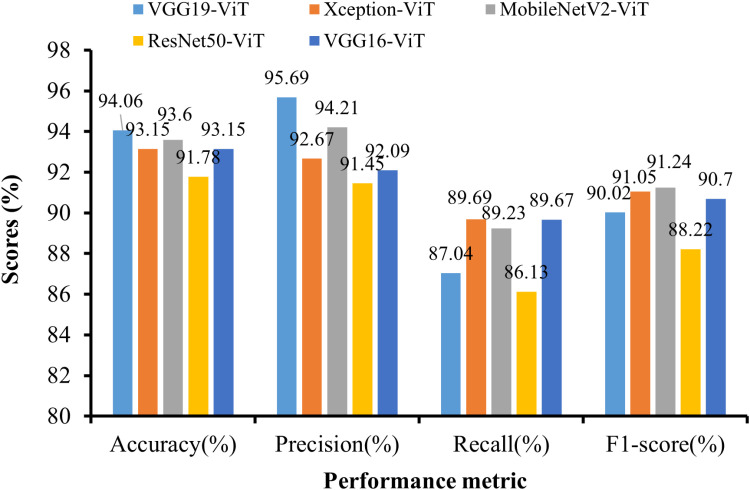
Graphical representation of TLM-ViT performance metrics.

Table 9 and Fig 12 present the performance analysis of the TLM-ViT (64-8-8) model across various TL architectures: VGG16, VGG19, Xception, MobileNetV2, and ResNet50, combined with ViT.

**Table 9:** Performance analysis of TLM-ViT.

Models	Accuracy	Precision	Recall	F1-score
VGG16-ViT	93.15	92.09	89.67	90.70
VGG19-ViT	94.06	95.69	87.04	90.02
Xception-ViT	93.15	92.76	89.69	91.05
MobileNetV2-ViT	93.60	94.21	89.23	91.24
ResNet50-ViT	91.78	91.45	86.13	88.22
Proposed CNN-ViT	**95.60**	**94.75**	**92.40**	**93.45**

**Fig 13:**
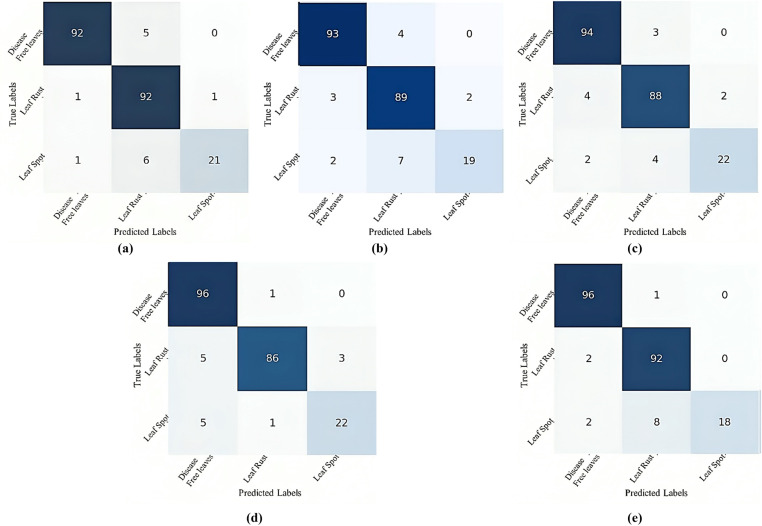
Confusion matrix of the TLM-VIT models: (a) MobileNetV2-ViT, (b) ResNet50-ViT, (c) Xception-ViT, (d) VGG16-ViT and (e) VGG19-ViT.

The metrics evaluated include accuracy, precision, recall, and the F1 score. Among the architectures, VGG19-ViT achieves the highest accuracy of 94.06% and a precision of 95.69%. MobileNetV2-ViT achieves the highest F1 score of 91.24%. Overall, these results indicate that different architectures combined with ViT exhibit varying strengths in terms of accuracy, precision, and computational efficiency, providing insights for selecting the appropriate architecture based on the specific requirements of the task at hand. The confusion matrices of the suggested model, which were tested using VGG16, VGG19, Xception, MobileNetV2, and ResNet50 combined with ViT, are displayed in Fig 13.

**Fig 14:**
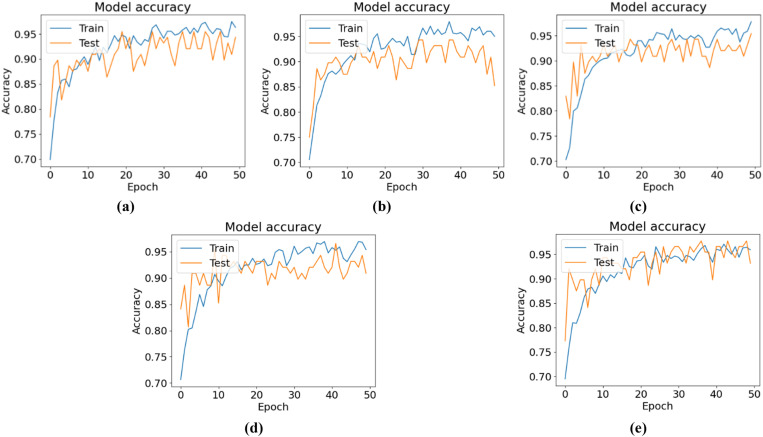
Accuracy curves of the TLM-VIT models: (a) MobileNetV2-ViT, (b) ResNet50-ViT, (c) Xception-ViT, (d) VGG16-ViT and (d) VGG19-ViT.

**Fig 15:**
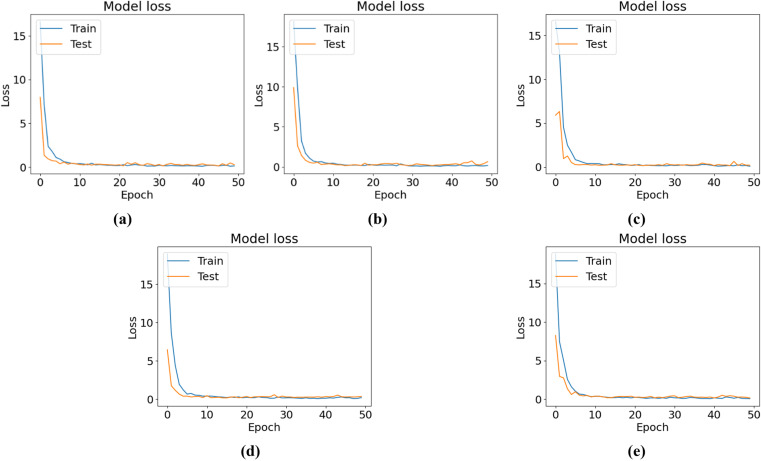
Loss curves of the TLM-VIT models: (a) MobileNetV2-ViT, (b) ResNet50-ViT, (c) Xception-ViT, (d) VGG16-ViT and (d) VGG19-ViT.

**Fig 16:**
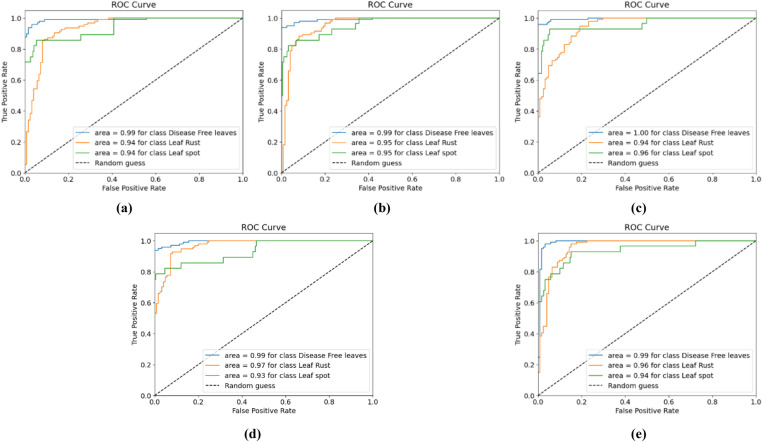
ROC curves of different TL-ViT models: (a) MobileNetV2-ViT, (b) ResNet50-ViT, (c) Xception-ViT, (d) VGG16-ViT and (e) VGG19-ViT.

The classification accuracies and loss curves of the training and testing sets for five ViT-TL models, VGG16, VGG19, Xception, MobileNetV2, and ResNet50, are displayed in Figs 14 and 15, respectively. The ROC curves for all five TLM-ViT models are presented in Fig 16.

### 4.5. Grad-CAM visualization

**Fig 17:**
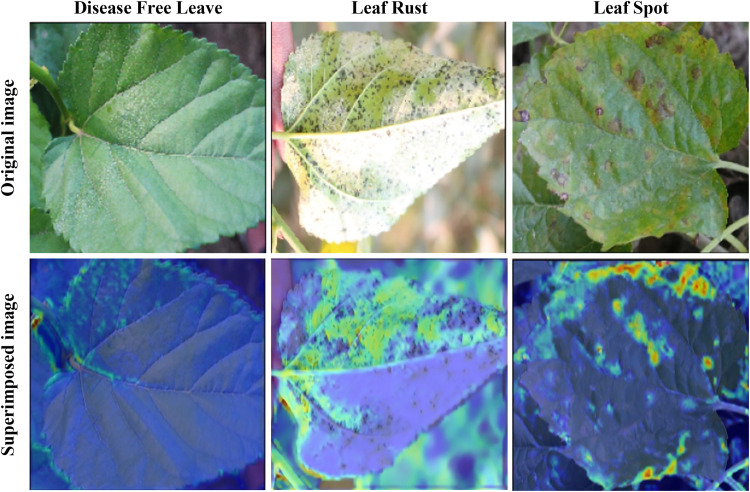
Correctly identified Grad-CAM visualization for different mulberry diseases.

The Grad-CAM visualization presented in Fig 17 offers insights into the model’s heatmap focus when identifying different leaf conditions. The images depict disease-free leaves, leaf rust, and leaf spots, both individually and superimposed with their corresponding heatmaps. By overlaying the attention maps onto the original images, we can discern the regions crucial for the model’s classification decision. This technique helps highlight areas of interest, providing valuable interpretability of the model’s decision-making process and potentially aiding in the identification and understanding of various leaf conditions in agricultural contexts. Furthermore, in the superimposed images of leaf spots and leaf rust, specific features are emphasized. In the leaf spot image, the spots are highlighted (regions without blue highlighting), allowing easier identification of affected areas. Similarly, in the leaf rust image, the rust areas are highlighted, aiding in the detection of rust-infected regions. Conversely, in the image depicting disease-free leaves, some edges are detected, indicative of healthy foliage, with no regions highlighted within the leaf, confirming its disease-free status. These distinctions provide additional clarity and insight into the model’s focus areas, enhancing the interpretability of its classification decisions.

Despite the model achieving decent accuracy in identifying disease-free leaves and leaf rust, the superimposed XAI images correctly identified affected and unaffected areas. Interestingly, even though the model demonstrated lower accuracy in detecting leaf spots, the superimposed XAI result was very effective in accurately identifying the spots. This finding indicates that the model’s effectiveness in highlighting relevant features is robust, even when the overall accuracy is lower. Thus, Grad-CAM visualization not only clarifies the model’s decision-making process but also underscores its ability to correctly identify key features of leaf conditions, increasing confidence in its practical application.

**Fig 18:**
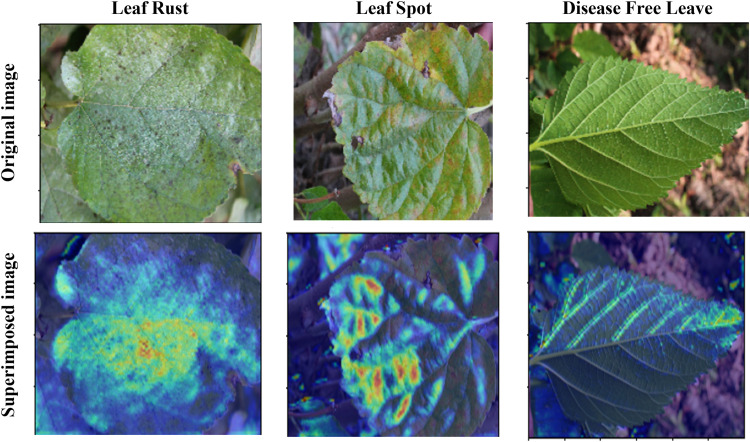
Misclassified Grad-CAM visualization for different mulberry diseases.

Fig 18 illustrates the Grad-CAM visualizations for instances where the proposed CNN-ViT model misclassified mulberry leaf diseases. In the first column, leaf rust disease was incorrectly classified as disease-free. The Grad-CAM heatmap indicates that the model focused on regions that did not exhibit significant rust spots. This suggests that the features of the model associated with leaf rust were either subtle or not sufficiently learned, leading to incorrect classification. In the second column, actual leaf spot disease was misclassified as disease-free. The Grad-CAM visualization shows that the model’s attention was not effectively drawn to the spots. The regions highlighted might have been insufficient to trigger positive identification of the leaf spot, indicating that the model might require further training to detect and differentiate these spots accurately. In the third column, the actual disease-free leaves were predicted to be leaf rust. The Grad-CAM heatmap reveals that the model focused on areas that have visual similarities between leaf spots and rust, such as discoloration or texture changes. This confusion indicates that the model has some difficulty distinguishing between these two diseases, which might share some overlapping visual characteristics.

### 4.6. Discussion

#### 4.6.1. Comparative analysis with transfer learning models.

The proposed CNN-ViT model outperforms the other pre-trained models, such as VGG16, VGG19, EfficientNetB3, and MobileNetV2, in terms of performance, as shown in Table 10. These results suggest that the pretrained models require additional fine-tuning and optimization to handle the specific task of mulberry leaf classification effectively. The performance gap between these models and the proposed CNN-ViT highlights the robustness of the proposed model, which has been optimized for tasks with better overall performance.

**Table 10:** Performance analysis of TLM models.

Models	Accuracy	Precision	Recall	F1-score
VGG16	88.70	87.95	85.50	86.70
VGG19	89.10	88.35	85.80	87.05
EfficientNetB3	90.45	89.50	87.60	88.50
MobileNetV2	91.20	90.35	88.10	89.20
Proposed CNN-ViT	**95.60**	**94.75**	**92.40**	**93.45**

#### 4.6.2. Comparative analysis with SOTA models.

Table 11 provides a comparative analysis between the proposed model and existing methods in the field of mulberry leaf disease detection. This dataset, which is relatively novel, has limited prior research. Nonetheless, the proposed CNN-ViT model demonstrates superior performance, achieving an accuracy of 95.43%, along with high precision, recall, and F1-score metrics.

**Table 11:** Comparison between the proposed model and other reported models.

Reference	Models	Accuracy (%)	Precision (%)	Recall (%)	F1-score (%)	XAI used	Model Complexity Analysis
Nahiduzzaman et al. [10]	PDS-CNN-WA	95.05	93.20	92.80	93.00	SHAP	Yes
Wen et al. [18]	INSE-ResNet50	**98.72**	**98.71**	**98.73**	**98.72**	Grad-CAM	Yes
Himanshu et al. [13]	capsule neural network	82.04	82.00	80.00	81.00	No	No
Thipwimon et al. [14]	Ensemble 5 pretrained models	94.75	–	–	–	No	No
Salam et al. [17]	Pretrained MobileNetV3 small	96.4	97.00	96.4	96.4	Grad-CAM	No
Rakesh et al. [15]	PNN	98.00	–	–	–	No	No
Proposed Model	CNN-ViT	95.60	94.75	92.40	93.45	Grad-CAM	Yes

The proposed CNN-ViT model demonstrated a balanced performance across all the leaf disease classes, achieving high F1-scores and recall rates even for minority classes such as leaf spots, which were often underrepresented in the dataset. This balanced performance ensured that the model did not overfit to the majority class, which is a common issue in many SOTA models that reported high overall accuracy but failed to generalize well across all classes. Most SOTA methods either balance their datasets artificially or use pre-trained models on large, well-balanced datasets. For example, Nahiduzzaman et al. [10] converted an imbalanced dataset into a balanced dataset by selecting 2000 samples from each class, which did not reflect real-world scenarios where the class distribution is naturally imbalanced. The proposed model, on the other hand, effectively handles the imbalanced dataset without artificially balancing the data. This is crucial for real-world applications where such imbalances are common, making the model more robust and better suited for practical deployment.

Another critical gap in many existing SOTA methods was the lack of interpretability. While models such as the capsule neural network and PNN focused purely on accuracy, they did not provide insights into how the model interpretability. In agriculture, where users need to trust AI systems, interpretability is crucial. The proposed model integrated Grad-CAM for explainability, allowing users to visualize the areas of the image that influenced the model’s decision. This feature significantly improved the practical applicability of the model, as it improved trust and transparency in the classification process, a factor that most SOTA methods do not address.

Additionally, Salam et al. [17] continued their experiments with a pretrained model, MobileNetV3 Small, which achieved 96.4% accuracy. However, they did not use any custom model of their own and achieved their results by balancing the dataset. Another problem in their recent work was that they used Grad-CAM, but the Grad-CAM results were poor because they did not detect any specific portions effectively. Several SOTA methods, such as INSE-ResNet50 [18], rely on highly complex architectures that, while achieving higher accuracy, result in significant computational overhead. These models are not optimized for real-time applications, which are essential in agriculture for real-time monitoring and decision-making. Wen et al.’s INSE-ResNet50 model achieved high accuracy by using highly specific configurations and additional datasets that may not be publicly accessible. The current model was designed to be scalable and applicable to real-world agricultural challenges without the need for extensive data preprocessing or augmentation, which could distort practical use cases. Additionally, they did not mention the time required to predict an image, which makes it challenging to compare their results.

While the proposed model might not outperform all the SOTA methods in terms of raw accuracy, it addresses several critical challenges, including its ability to perform well in an imbalanced class situation, which is common in real-life scenarios and interpretability. These factors make the proposed model suitable for agricultural domain, where scalability, interpretability, transparency, and efficiency are equally important as the accuracy.

#### 4.6.3. Strengths, limitations and future work.

The main strengths of this research are that the proposed CNN-ViT model demonstrated ability to extract deep features, which helped in producing good accuracy in classifying mulberry leaf diseases even an imbalanced dataset. Furthermore, it was demonstrated that the model can learn and generalize better than the TL models. The proposed model integrated XAI techniques, specifically Grad-CAM, clearly highlighting the regions of interest that indicated the characteristics of a particular leaf disease, which helped the decision-making process more transparent and understandable by the farmers. This is crucial for practical applications in scenarios requiring real-time analysis, such as monitoring the health of crops in the field. Another Advantage of the proposed model is that it can classify the image type very quickly which is ideal for edge devices.

The CNN + ViT hybrid approach offers a more efficient architecture by reducing the number of trainable parameters, leading to faster training and better feature selection. By using a CNN for initial feature extraction, it minimizes redundant computations and allows ViT to focus on high-level contextual relationships. In contrast, direct ViT models require significantly more parameters due to the full self-attention mechanism applied across numerous image patches, leading to higher computational costs. Additionally, direct ViT models typically demand large datasets for effective training, as they lack the inductive biases of CNNs, making them less efficient for small or imbalanced datasets.

While the proposed model demonstrated strong overall performance, the ‘Leaf Spot’ class presented lower recall and F1-score metrics, most likely due to class imbalance and visual similarities between leaf diseases. This finding indicated that the model had difficulty distinguishing between certain disease types, particularly in underrepresented classes.

Although the present study has made significant progress in mulberry leaf disease detection via a CNN-ViT model, there are several avenues for future exploration and improvement. While Synthetic Minority Oversampling Technique (SMOTE) and similar synthetic data generation methods offer potential, their effectiveness may vary depending on the dataset and model architecture. The importance of investigating other techniques, such as GAN-based synthetic data generation, to assess their impact on model performance could be evaluated in the future, particularly for minority classes such as “Leaf Spot,” to determine the most effective method for optimizing performance. Future work will focus on getting better accuracy with a very lightweight model by further optimizing the model. Future work could be focused on further validating the applicability of the model through exploring additional datasets. Finally, continued collaboration with domain experts in agriculture and ML researchers can facilitate the refinement and validation of the proposed approach, ultimately contributing to the practical adoption and impact of automated disease detection technologies in sericulture and beyond.

#### 4.6.4. Practical significance.

The practical significance of this research could potentially transform the monitoring and management of mulberry crops, crucial agricultural resources for the silk industry. The CNN-ViT model could be effectively generalized to broader agricultural applications, enhancing its impact in the field. By adapting the model to detect diseases in other crops, such as rice, wheat, and tomatoes, it can address a wide range of plant diseases that are visually identifiable through image classification techniques. Transfer learning can enable the model to retain core image features while fine-tuning it for specific crops and their unique disease characteristics. Furthermore, integrating this model into precision agriculture systems, such as drones collecting real-time data, can automate crop monitoring, ensuring early detection of diseases and minimizing crop loss. The ability to quickly and accurately identify leaf conditions can significantly enhance crop health monitoring, leading to timely interventions that prevent the spread of diseases. This not only improves crop yield and quality but also reduces the reliance on manual inspections, saving time and resources.

Exploring how well non-expert users, such as farmers, can interpret Grad-CAM visualizations would provide crucial insights into the real-world utility of the model. When assessing healthy leaves, Grad-CAM typically shows a uniform or cool-toned map (blue or green), suggesting minimal to no disease indicators. The absence of highlighted regions informs farmers about healthy leaf conditions. For rust-infected leaves, the Grad-CAM heatmap often highlights rusty or reddish-brown patches, which are seen by farmers as warm colors (such as yellow, orange, or red), with a focus on specific rusted areas. In cases of leaf spot disease, Grad-CAM visualizations highlight circular or irregular patches, and farmers observe areas with relatively high concentrations of warm colors around these spots, indicating the presence of the disease. Upon providing appropriate training to farmers who do not have much technical knowledge, the developed system can help them understand and compare the appearance of different leaf diseases based on the location and density of highlighted areas with different colors. This research, therefore, contributes to efficient, sustainable, and informed agricultural practices, benefiting both farmers and the broader agricultural community.

## 5. Conclusions

In conclusion, this study demonstrated the efficacy of an AI model that combined ViT with a custom CNN architecture for mulberry leaf disease recognition in a multi-class classification with the ability to handle imbalanced data. Through systematic hyperparameter tuning and experimentation, the proposed model was selected, leading to high accuracy, precision, and computational efficiency. The best results were achieved with the ViT configurations of 64 (projection dimension), 8 (sum of heads in attention mechanism), and 8 (transformer layers), which yield accuracy of 95.60%, precision of 94.75%, recalls of 92.40%, and F1-scores of 93.45%. The proposed model also outperformed other popular transfer learning architectures such as VGG16, VGG19, Xception, MobileNetV2, and ResNet50, combined with ViT or without ViT. Additionally, the affected regions were detected via Grad-CAM, providing easy explainability of the model’s decisions for the end users.
